# Use of targeted SNP selection for an improved anchoring of the melon (*Cucumis melo* L.) scaffold genome assembly

**DOI:** 10.1186/s12864-014-1196-3

**Published:** 2015-01-22

**Authors:** Jason M Argyris, Aurora Ruiz-Herrera, Pablo Madriz-Masis, Walter Sanseverino, Jordi Morata, Marta Pujol, Sebastián E Ramos-Onsins, Jordi Garcia-Mas

**Affiliations:** IRTA, Centre for Research in Agricultural Genomics (CRAG) CSIC-IRTA-UAB-UB, 08193 Barcelona, Spain; Departament de Biologia Cellular, Fisiologia i Immunologia, Universitat Autònoma de Barcelona, Campus UAB, 08193 Barcelona, Spain; Institut de Biotecnologia i Biomedicina (IBB), Universitat Autònoma de Barcelona, Campus UAB, 08193 Barcelona, Spain; Centre for Research in Agricultural Genomics (CRAG) CSIC-IRTA-UAB-UB, 08193 Barcelona, Spain; Present Address: Sequentia Biotech, Campus UAB - Edifici CRAG, Bellaterra - Cerdanyola del Vallès, 08193 Barcelona, Spain

**Keywords:** Melon, SNP, Genome, Scaffold, Pseudomolecules, FISH, Karyotype

## Abstract

**Background:**

The genome of the melon (*Cucumis melo* L.) double-haploid line DHL92 was recently sequenced, with 87.5 and 80.8% of the scaffold assembly anchored and oriented to the 12 linkage groups, respectively. However, insufficient marker coverage and a lack of recombination left several large, gene rich scaffolds unanchored, and some anchored scaffolds unoriented. To improve the anchoring and orientation of the melon genome assembly, we used resequencing data between the parental lines of DHL92 to develop a new set of SNP markers from unanchored scaffolds.

**Results:**

A high-resolution genetic map composed of 580 SNPs was used to anchor 354.8 Mb of sequence, contained in 141 scaffolds (average size 2.5 Mb) and corresponding to 98.2% of the scaffold assembly, to the 12 melon chromosomes. Over 325.4 Mb (90%) of the assembly was oriented. The genetic map revealed regions of segregation distortion favoring SC alleles as well as recombination suppression regions coinciding with putative centromere, 45S, and 5S rDNA sites. New chromosome-scale pseudomolecules were created by incorporating to the previous v3.5 version an additional 38.3 Mb of anchored sequence representing 1,837 predicted genes contained in 55 scaffolds. Using fluorescent in situ hybridization (FISH) with BACs that produced chromosome-specific signals, melon chromosomes that correspond to the twelve linkage groups were identified, and a standardized karyotype of melon inbred line T111 was developed.

**Conclusions:**

By utilizing resequencing data and targeted SNP selection combined with a large F2 mapping population, we significantly improved the quantity of anchored and oriented melon scaffold genome assembly. Using genome information combined with FISH mapping provided the first cytogenetic map of an *inodorus* melon type. With these results it was possible to make inferences on melon chromosome structure by relating zones of recombination suppression to centromeres and 45S and 5S heterochromatic regions. This study represents the first steps towards the integration of the high-resolution genetic and cytogenetic maps with the genomic sequence in melon that will provide more information on genome organization and allow for the improvement of the melon genome draft sequence.

**Electronic supplementary material:**

The online version of this article (doi:10.1186/s12864-014-1196-3) contains supplementary material, which is available to authorized users.

## Background

Melon (*Cucumis melo* L.) is a highly diversified species that is cultivated worldwide, with more than 31 million tons produced in 2011 (http://faostat.fao.org). It is a eudicot diploid species (2n = 2x = 24) that belongs to the Cucurbitaceae family which includes other important vegetable crops such as cucumber, watermelon and squash. In recent years melon has become a model system for studying important biological processes as plant sex determination [1,2], phloem transport [3], and fruit ripening [4]. At the same time, several genetic and genomic tools are available in melon, such as saturated genetic maps [5] or EST databases [6].

The advent of high throughput next generation sequencing (NGS) technologies combined with declining cost has led to an explosion in the number of sequenced plant genomes in the past several years [7]. The majority have used a whole genome shotgun (WGS) strategy and a hybrid approach to genome assembly, incorporating both short reads typically generated by NGS platforms, and Sanger-derived end sequences from large insert BAC and fosmid clones [8]. The genome of melon was recently sequenced, producing a genome assembly with N50 scaffold size of 4.68 Mb and N90 index of 78, of good quality when compared with other plant genomes recently reported based on NGS [9]. The quality of the genome assembly has an impact on applications of the genome sequence by providing, among others, a reference genome for resequencing analysis. Comparisons of resequenced individuals to a reference genome offer a means to identify and characterize genetic polymorphisms, of which single nucleotide polymorphisms (SNPs) are the most important and abundant. SNP discovery using NGS approaches has been reported in many crop species [10], and it has been described that SNPs identified through whole genome resequencing have a low false discovery rate compared to other methods [11]. The abundance of SNPs in the genome, coupled with the diversity of technologies for performing multiplex assays that can range from genotyping single SNPs at a time to over one million in parallel [12], make them a powerful tool for genetic mapping and marker assisted breeding.

The assembly of genomic scaffolds to generate chromosome-scale sequences, or pseudomolecules (PM), is done by integrating them with genetic or physical maps [13]. A genetic map of sufficient accuracy and marker density is therefore essential for anchoring genomic scaffolds to linkage maps. High density genetic maps developed with single sequence repeat (SSR) and SNP markers have been used in many crop species to anchor genome assemblies. Recent examples include peach (*Prunus persica*) [14], diploid strawberry (*Fragaria vesca*) [15] and apple (*Malus x domestica*) [16] as well species of the Cucurbitaceae family such as watermelon (*Citrullus lanatus*) [17], and cucumber (*Cucumis sativus*) [18]. A set of 602 polymorphic SNPs, derived from a *C. melo* expressed sequence tag (EST) collection [19] were used to produce a high resolution genetic map based on 72 doubled haploid lines (DHLs) of the cross “Piel de Sapo” (PS) [T111] x Songwhan Charmi’ (SC) [PI 161375] to anchor and orient 87.5% (316.3 Mb) and 80.8% (291.9 Mb) of the melon scaffold genome assembly v3.5, respectively [9].

Karyotype analysis in *Cucumis* is difficult due to the small size and poor stainability of chromosomes [20]. Despite this, karyotypes based on chromosome banding and morphology have been developed for several melon types with varying results [21-23]. Fluorescence in situ hybridization (FISH) allows direct mapping of both single-copy and repetitive DNA sequences on chromosomes, and is a more robust and reliable method of identifying chromosomes and establishing an accurate karyotype. FISH is an important tool not only in cytogenetics, but also in genomic applications to estimate the size and positions of gaps in genome assemblies following sequencing, and in characterizing regions of recombination suppression where the resolution of genetic linkage maps are insufficient [24]. Probes commonly used in FISH karyotyping in plant species are the 45S and 5S rRNA genes, tandemly repeated sequences near telomeres, centromere-specific repeats, and large insert DNA clones such as bacterial artificial chromosomes (BACs), yeast artificial chromosomes (YACs), and fosmids [25]. Extensive cytogenetic studies have been conducted using FISH with these probes in cucumber, including the development of integrated molecular cytogenetic maps [26-28]. Relatively less has been done in melon, but several studies using FISH with 45S, 5S, and centromere specific probes have been performed to develop karyotypes [29-31]. Cross species FISH, based on sequence similarity of probes between closely related species, has been used with cucumber fosmids to make inferences on melon chromosome structure and karyotype [32-34]. However, in some instances, fosmids mapped to several non-target melon chromosomes, or failed to map. Thus, the importance of FISH in genomic applications, and non-target specific hybridization using cross-species FISH in melon, highlights the need for an efficient FISH methodology using melon specific large insert DNA probes.

Although the percentage of assembly effectively anchored to chromosomes of the melon genome described in [9] was of a comparable level to some recently sequenced crop species [16,17], insufficient marker coverage in the genetic map left several large, gene-rich scaffolds unanchored. Also, due to the small population size and insufficient recombination, many anchored scaffolds contained just one mapped marker, or contained multiple tightly linked markers whose map order was undefined, which made orientation of some scaffolds impossible. To address these deficiencies, we detail the use of resequencing data between of PS and SC to develop a new, better distributed set of SNP markers, and in combination with a larger segregating population, a high-density genetic map to augment the amount of anchored and oriented melon genome scaffolds. Also, we describe the construction of more complete PMs required to accurately represent the scaffold genome assembly. We also describe the use of BAC probes to identify the 12 melon chromosomes and in combination with two color FISH, orient them with respect to the genetic map and PMs. This is an important first step in developing an integrated molecular cytogenetic map which will be a key tool in improving the quality of the draft genome of melon for future uses.

## Results

### SNP validation and construction of the genetic map

**Figure 1:**
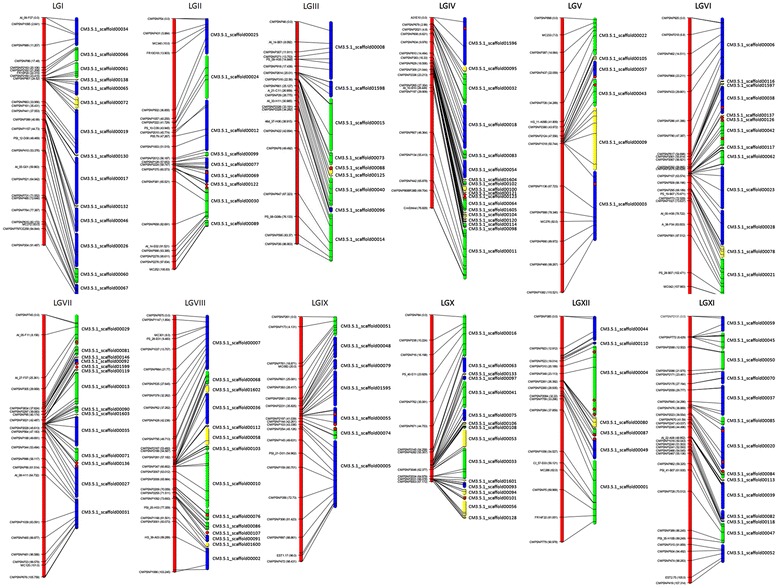
**Anchoring of the melon scaffold genome assembly to the PS x SC F2 genetic map.** Red bars represent the 12 melon linkage groups; SNPs are located according to genetic distance (cM). Melon genome scaffolds were positioned in each linkage group with corresponding genetic markers. Blue, scaffolds in positive orientation; green, scaffolds with negative orientation (reverse and complemented); yellow, scaffolds that were anchored but not oriented. Red dots represent locations of centromere-specific repeats inferred by *in silico* analysis.* Not all SNP names are represented in the genetic map.

Melon genome assembly v3.5 is composed of 1,599 scaffolds, of which 87 were previously anchored to the genetic map, representing 87.5% of the scaffold assembly [9]. As 98.5% of the scaffold assembly is contained in the 150 largest scaffolds (Additional file 1: Table S1) and SNPs between PS and SC lines are available [9], we mined SNPs from 147 of the 150 scaffolds to construct a new genetic map in an F2 population derived from the PS x SC cross. The scaffolds ranged in size from 9.16 Mb (CM3.5_scaffold00004) to 31.5 kb (CM3.5_scaffold00144). Three scaffolds (CM3.5_scaffold00134, 147, and 150) were excluded due to the inability to extract high-quality SNPs from the genome sequence. 768 SNPs were selected for the design of a GoldenGate assay, 435 SNPs from unanchored scaffolds (sub-set one) and 333 SNPs from already anchored scaffolds (sub-set two) (Additional file 1: Table S2). Overall, 599/768 (78%) of SNPs were successfully genotyped in the F2 population. Of the 435 new SNPs comprising sub-set one, 288 (66%) were validated. The remaining 147 SNPs from this set either failed to amplify, presented fused or extra clusters (null alleles), or were false SNPs. The average designability rank score for failed SNPs (0.86) was markedly lower compared to that for successful SNPs (0.95). Fifty percent of failed SNPs (74/147) were designed from the 31 smallest scaffolds that had a mean size of 121 kb. Therefore, SNPs that were designed to just 1/5 of the 147 scaffolds originally selected for re-anchoring the scaffold genome assembly accounted for half of the failure rate. The success rate for the second, previously validated sub-set of SNPs was much higher, with 311 of 332 (94%) functioning. The successful SNPs from the two sub-sets were combined into a single set of 599 SNPs that was entered into JoinMap for construction of the genetic map. Nineteen SNPs were excluded from the mapping process due to extreme segregation distortion, missing data, or implausible fit in a linkage group (LG). Following this step, a core set of 580 SNPs (279 from set one + 301 from set two) and 139 PS x SC F2 lines were used to construct a high resolution genetic map spanning 1,153 cM of the 12 melon LGs with a mean of 49 SNPs per LG (Figure 1, Table 1). The size of LGs ranged from 113.6 cM (LG6) to 67.2 cM (LG10) with the number of SNPs per LG ranging from 29 to 65. Average spacing of markers genome-wide was 1.99 cM/SNP.

**Table 1:** **Anchoring of melon genome assembly v 3.5.1 to the PS x SC F2 genetic map**

**LG**	**LG size(cM)**	**SNPs used in map construction**	Scaffolds anchored*	**Genome anchored (bp)**	**% Scaffold assembly**	**Oriented scaffolds**	**Genome oriented (bp)**	**% Scaffold assembly**	**Recombination rate (cM/Mb)**
I	91.5	53	14	35,370,099	9.8	11	35,197,227	9.7	2.6
II	100.8	40	9	26,185,771	7.2	8	26,070,550	7.2	3.9
III	86.8	39	9	29,379,469	8.1	7	28,223,253	7.8	3.0
IV	76.8	65	18	33,106,231	9.2	10	30,831,195	8.5	2.3
V	110.5	29	6	28,332,775	7.8	4	19,742,536	5.5	3.9
VI	113.6	54	13	35,927,859	9.9	7	33,605,504	9.3	3.2
VII	105.8	57	14	26,760,857	7.4	9	26,066,430	7.2	4.0
VIII	103.2	56	14	32,500,408	9.0	9	28,100,052	7.8	3.2
IX	98.4	39	7	24,101,567	6.7	7	24,101,567	6.7	4.1
X	67.2	56	16	25,347,316	7.0	8	18,851,255	5.2	2.7
XI	107.3	60	14	31,429,130	8.7	13	31,190,979	8.6	3.4
XII	91	32	7	26,394,393	7.3	6	25,034,488	6.9	3.8
**Total**	**1162**	**580**	**141**	**354,835,875**	**98.2**	**99**	**327,015,036**	**90.5**	**3.3**

*Includes newly created scaffolds (see text).

In constructing the genetic linkage map, major areas of segregation distortion (p < 0.05) were evident on LGs I and IV spanning 41.4 and 38 cM, respectively (Additional file 1: Table S3). In both cases, marker alleles were skewed toward the SC parent. Segregation distortion was reported previously in DHL and F2 mapping populations of melon derived from the PS x SC cross [35,36] but did not correspond to the regions detected in this study. Conversely, segregation distortion was not detected in backcross populations used for NIL development [37].

### Genome re-anchoring

The genetic map was used to perform a re-anchoring of the scaffold genome assembly to the 12 LGs (Figure 1, Table 1). By anchoring the genetic map, six chimeric scaffolds (CM3.5_scaffold00022, CM3.5_scaffold00029, CM3.5_scaffold00045, CM3.5_scaffold00053, CM3.5_scaffold00063, CM3.5_scaffold00072), each mapping in two different locations in the genome (LGV/LGVIII, LGVII/X, LGVIII/LGXI, LGVII/LGX, LGIV/LGX, LGI/LGIV, and LGVII/LGX, respectively) were detected (Additional file 1: Table S4). These were due to single misassemblies within scaffolds where paired-end links between contig sequences were erroneously joined during the genome assembly. After splitting, newly created scaffolds were designated as CM3.5.1_scaffold01600-1605. Additionally, a misassembly was identified in CM3.5_scaffold01599. CM3.5_scaffold00056 was first detected as chimeric in assembly v3.4, and split into CM3.5_scaffold01599 for v3.5 [9]. As the split was likely not performed properly, 91.6 kb was trimmed from the latter and appended to the former. The melon genome assembly was updated to v3.5.1, identical to v3.5 except for the above modifications and a slightly decreased final size of the assembled genome (375.47 Mb).

**Figure 2:**
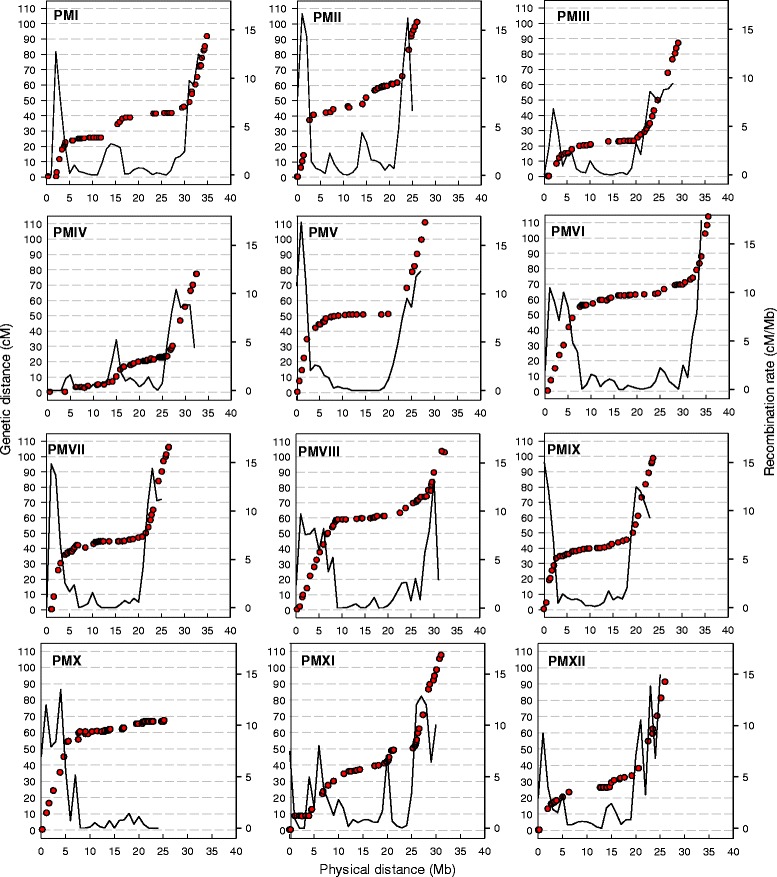
**The ratio between genetic and physical distances and recombination frequency of the 12 melon pseudomolecules.** For each SNP marker (filled red circle) in the PS x SC F2 genetic map, the genetic distance in centimorgans (cM) is plotted according to its physical position in megabases (Mb) on the pseudomolecule (PM). Recombination (solid line) rate was plotted in 1 Mb sliding intervals (see methods).

Each of 141 scaffolds (135 from assembly v 3.5 + 6 newly created from chimeras), containing over 354.84 Mb, or 98.2% of the scaffold genome assembly, was anchored to the genetic map with a minimum of 1 and a maximum of 12 SNPs (Additional file 1: Table S2) The number of scaffolds anchored per LG ranged from 6 (LGV) to 18 (LGIV) with a size ranging from 9.16 Mb to 41 kb. The size of newly anchored scaffolds ranged from 4.17 Mb (CM3.5.1_scaffold00036) to 41.2 kb (CM3.5.1_scaffold00146) with an average size of approximately 730 kb. We were able to anchor 99 scaffolds with 2 or more SNPs and thus determine orientation for over 327 Mb (90.5%) of the scaffold assembly. Forty-two scaffolds, totaling 27.8 Mb, remained unoriented (Additional file 1: Table S5). Furthermore, the ordering of 20 of the unoriented scaffolds, located on six PMs (III, IV, VI, VII, VIII, and X) was uncertain. Most of these were less than 1 Mb in size and were anchored in recombination suppression regions (Figures 1 and 2). Although the average marker spacing was 694 kb/SNP, large gaps were present on the physical map; e.g. 6.4 Mb in CM3.5.1_scaffold00004 on LGXII between CMSNP348 and CMPSNP211 and a 5.1 Mb gap in CM3.5.1_scaffold00017 on LGI between CMPSNP1111CE74 and CMPSNP399. We failed to anchor 12 scaffolds targeted from assembly v3.5 containing 1.52 Mb of sequence ranging in size from 410–31 kb with at least 1 SNP marker (Additional file 1: Table S1, Additional file 1: Table S2). Anchored scaffolds were assembled into 12 PMs according to the nomenclature and established orientation of the melon LGs of the consensus linkage map [5]. These were supplemented by a virtual chromosome 0 containing 20.6 Mb of sequence. The PMs ranged in size from 35.9 (LGVI) to 24.1 (LGIX) Mb and the number of newly anchored scaffolds ranged from 0 (PMIX) to 11 (PMX) per PM (Figure 1, Table 1, Additional file 1: Table S5).

The genome-wide recombination rate (cM/Mb) calculated for the melon genome was 3.3 cM/Mb. The recombination rates among PMs varied widely from a minimum of 2.3 cM/Mb on LGIV, to 4.1 cM/Mb on LGIX (Table 1) and, with the exception of PMX, was correlated to physical size, with the shortest chromosomes exhibiting higher recombination rates, and vice versa. The ratio between genetic and physical distances localized regions of high recombination mostly concentrated around the ends of the PMs, with recombination suppression increasing as distance from the ends increased (Figure 2, Additional file 1: Table S6). An exception was PMX, which showed high recombination only on one end of the chromosome, and a large zone of recombination suppression extending for approximately 16 Mb to the opposite end. Recombination rates within chromosomes also varied widely, reflective of the size and distribution of zones. BLAST analysis with four distinct centromere specific repeats: sSat107 and CentSpA, B, C as well as repeats specific to the nucleolus organizer region (NOR) or 45S rDNA and 5S rDNA sites, corresponded with zones of recombination suppression (Figure 1, Additional file 1: Table S7, Additional file 1: Table S8).

### Karyotype of PS

**Figure 3:**
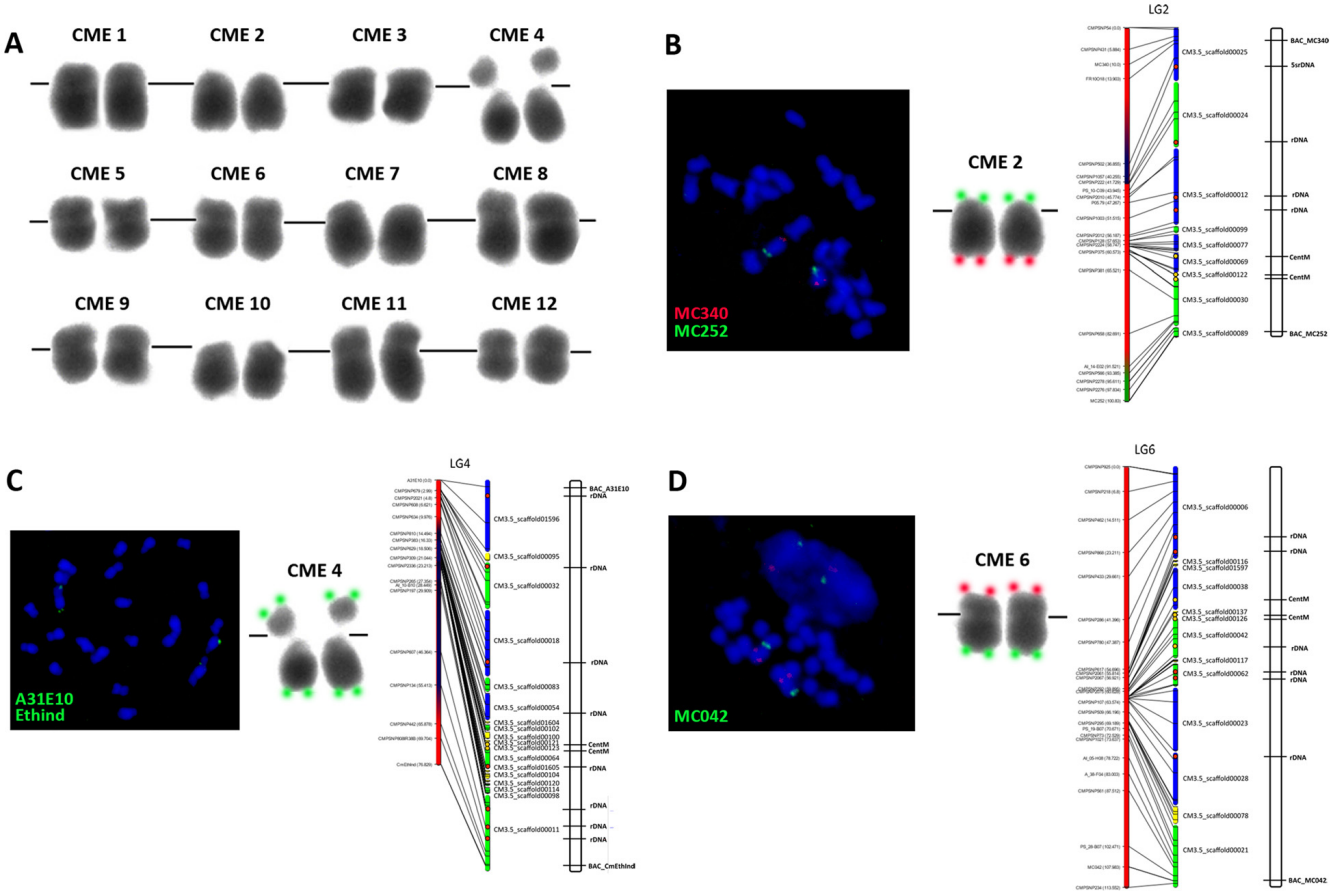
**Standarized karyotype of the 12 PS melon chromosomes.** Karyotype of PS **(A)** and 2 color FISH with BAC probes and ideograms for location of centromere specific, 45S, and 5S repeats identified by BLAST for CME2 **(B)**, CME4 **(C)** and CME6 **(D)**.

Nineteen single copy BACs were used to identify the 12 metaphase chromosomes of melon designated CME 1–12 (Table 2, Figure 3A). Chromosomes were then assigned to their corresponding LGs according to the map positions of 19 genetic markers (13 RFLP, 6 SNP) contained in these BACs. Seven chromosomes (CME 2, 4–7, 9 and 11) were labeled with 2 BAC probes located in ends of each chromosome, allowing the identification of the short (p) and long (q) arms, and orientation of chromosomes (Table 2, Additional file 2: Figure S1). Single BACs were hybridized to the long arms of CME 1, 3, and 12 and to the short arm of CME 10. The designation for CME 8 was uncertain. Chromosomes were categorized morphologically as metacentric (CME 12), sub-metacentric (CME 1, 3, 4, 5, 6, 8, 9, 11) or acrocentric (CME 2, 7, 10) and a standardized melon karyotype constructed (Table 2, Figure 3A). The relative sizes of chromosomes was visible in the karyotype, with CME 9, 10 and 12 being the smallest and most compact, while CME 1, 4, and 11 were among the largest. The structure of PMs corresponded to the morphology of CME 2, 4, and 6 (Figure 3B-D). Centromere positions indicated by pSat107 and CentSp sequences correlated well to zones of recombination suppression visible in the karyotype, and to the presence of many, relatively small scaffolds on the physical map. A large constriction was evident on CME4 (Figure 3C). FISH mapping of BAC clones on chromosomes confirmed the correct positioning of genomic scaffolds within PMs as the hybridization signals were located on the extremes of chromosome arms, as predicted by their anchoring to the genetic map (Figure 3B-D).

**Table 2:** **Markers and BAC clones used to identify and orient the 12 melon chromosomes**

**Chr**	**LG**	Marker*	**Scaffold**	**Scaffold position (bp)**	**BAC clone**	**Chr arm**	**Chr form**
CME1	I	MC216	CM3.5scaffold00034	434,148 - 433,875	39G23	q-arm	sm
CME2	II	MC252	CM3.5scaffold00089	58,157 - 57,875	27K12	p-arm	ac
		MC340	CM3.5scaffold00025	1,148,058 - 1,146,502	2N3	q-arm	
CME3	III	MC127	CM3.5scaffold00008	924,766 - 924,972	5P10	p-arm	sm
CME4	IV	CmEthImd	CM3.5scaffold00011	486,913 - 486,496	14C18	p-arm	sm
		A13E10	CM3.5scaffold01596	644,885 - 645,310	41C15	q-arm	
CME5	V	MC233	CM3.5scaffold00022	4,179,585 - 4,180,293	58B10	p-arm	sm
		MC276	CM3.5scaffold00003	6,499,792 - 6,499,614	01N03	q-arm	
CME6	VI	MC042	CM3.5scaffold00021	551,528 - 550,701	20H14	p-arm	sm
		MC069	CM3.5scaffold00006	671,768 - No hits	24I09	q-arm	sm
CME7	VII	MC125	CM3.5scaffold00031	3,442,638 - 3,442,244	12H24	p-arm	ac
		MC373	CM3.5scaffold00029	3,679,447 - 3,768,638	49P09	q-arm	
CME8	VIII	F080	CM3.5scaffold00007	305,608 - 306,861	22K19	--	sm
CME9	IX	MC092	CM3.5scaffold00051	1,034,849 - 1,035,091	24H03	p-arm	sm
		EST1.17	CM3.5scaffold00005	No hits - 8,239,117	53P08	q-arm	
CME10	X	CmEXP3	CM3.5scaffold00041	2,803,910 - 2,803,572	54E01	p-arm	ac
CME11	XI	MC326	CM3.5scaffold00045	1,714,833 - 1,715,049	51H20	p-arm	sm
		EST2.75	CM3.5scaffold00052	1,878,167 - 1,877,623	33O17	q-arm	
CME12	XII	MC286	CM3.5scaffold00001	2,781,343 - 2,783,316	20F17	q-arm	m

*From reference [49].

The chromosome (chr) form was assigned according the centromere position: acrocentric (ac), submetacentric (sm) and metacentric (m).

## Discussion

The resequencing of PS and SC, the parents of the DHL92 melon reference genome, identified 2.1 million putative SNPs occurring with a frequency of one per 176 bp [9]. We took advantage of this resource to identify a new set of 435 polymorphic SNPs between PS and SC to develop a high-resolution genetic map and anchor 354.98 Mb of sequence contained in 141 scaffolds, representing 98.2% of the ~361 Mb melon scaffold genome assembly v3.5.1 (Table 1). We oriented 325.4 Mb of sequence, representing 90.3% of the assembly.

The quantity of anchored, and especially oriented, genome was comparable to other recently sequenced crop plants [14-18,38-45] (Additional file 1: Table S9) and highest among sequenced cucurbit species [17,18]. It also represented a significant improvement from the anchoring of assembly v3.5 [9]. As SNPs used to construct the genetic map and anchor assembly v3.5 were selected at random from a collection of melon ESTs, their chromosomal location in the assembly was unknown [19]. Furthermore, SNPs derived from ESTs have a high false discovery rate compared to other sources as previously described in [11]. Therefore, marker coverage of the genome was likely incomplete because some scaffolds were either not represented by ESTs and subsequently, SNPs; the SNPs failed because they were false; or they failed to be validated for various other reasons as was seen in this study (Additional file 1: Table S1) and previously [19]. An additional problem was that SNP selection was biased to larger scaffolds, as none smaller than 575 kb were anchored in v3.5. We addressed these deficiencies and achieved a high level of anchoring first, by utilizing the resequencing data and the SNP calling pipeline, combined with an “inverse mapping” strategy of planned genetic map construction. As opposed to creating a genetic map at random, the inverse mapping strategy consisted of targeted SNP selection using data from the melon genome *a priori* to identify 147 of 150 largest scaffolds comprising the N98 index (Additional file 1: Table S1) and focusing SNP discovery to these scaffolds. This permitted SNP development for large and smaller scaffolds equally well. Second, to maximize the probability of orientation, multiple SNPs were chosen at the extremes of targeted scaffolds for the GoldenGate genotyping assay to maximize the physical distances between them. This strategy was combined with a doubling of the mapping population size to 139 F2 individuals to increase the probability of recombination, and subsequent orientation of the genomic scaffolds in the relation to the genetic map. In this way, both large, gene rich scaffolds (e.g. CM3.5.1_scaffold00036, 38, and 50) which accounted for most of the newly anchored sequence, as well as many smaller scaffolds less than 1 Mb in size could be anchored and oriented (Figure 1, Additional file 1: Table S5). A similar strategy of employing resequencing data for SNP discovery combined with a larger population was used to improve the anchoring and orientation of the soybean genome [44]. One disadvantage to choosing markers principally from the extremes of scaffolds was uneven marker distribution, and the presence of several large physical gaps between SNPs, e.g. on LGI and LGXII. This drawback was balanced by the precision of SNP selection and effectiveness of the genome anchoring and orientation using the inverse mapping approach, and should be an effective strategy for anchoring other plant genomes given the availability of a high quality reference genome and resequencing data from parental lines.

We constructed PMs of scaffold genome assembly v3.5.1 corresponding to the 12 melon chromosomes that were significantly more complete than v3.5, incorporating an additional 38.3 Mb of anchored sequence representing 1,837 predicted genes contained in 55 scaffolds. An additional 33.5 Mb of sequence was newly oriented. Compared to assembly v3.5, the 12 PMs were augmented by a mean of 3.2 Mb, and a maximum of 9.1 Mb in the case of LGX, together representing 25,065 (91.4%) of the predicted 27,427 melon genes. The quality of the anchoring of scaffold genome assembly v3.5.1, and subsequent construction of PM builds was verified by comparing it to assembly v3.5 [9]. The scaffold order was almost completely conserved, with some exceptions being inversions of adjacent scaffolds on PM IX (CM3.5.1_scaffold00055 and CM3.5.1_scaffold00074) and PM XI (CM3.5.1_scaffold00059 and CM3.5.1_scaffold00045) and changes in the orientation of others. The anchoring of the 55 new scaffolds to their corresponding LGs, and their ordering within PMs, was also largely verified with data from another melon genetic map (Nurit Katzir, personal communication). This provided a further independent measure of the quality and accuracy of the anchoring of assembly v3.5.1, for example, by confirming the new position of CM3.5.1_scaffold00078 on LGVI. Lastly, by adding markers to regions of scaffolds that were previously without them, the new genetic map helped to verify the integrity of existing scaffold builds, as well as improving the quality of the assembly through the identification of six mis-assemblies and correction in the size of two others (Additional file 1: Table S4).

While pseudomolecule build v 3.5.1 was significantly more complete compared to v 3.5, approximately 5.4 Mb of sequence contained in 1,464 scaffolds ranging from 2–20 kb in size and containing 448 genes remained unanchored. Despite this, expending the effort to anchor smaller scaffolds beyond those in the N98 index would have been prohibitive compared to the benefit (Additional file 3: Figure S2). Another feature of the new PM build was uncertainty in order and orientation of many mostly small scaffolds, principally in large zones of recombination suppression on LGIV, VI, and X. Recombination suppression zones are usually heterochromatic and gene poor, typically containing a high percentage of repeat sequences [46]. To anchor remaining scaffolds, and resolve uncertainties in order and position, above all, in zones of recombination will require the development of a FISH protocol on meiotic chromosomes. This will facilitate the comparison between the molecular and cytogenetic map as has been performed in cucumber [27], but only partially in melon [32]. In spite of these drawbacks, PM builds of v3.5.1 will be indispensable as a more complete reference sequence for comparative mapping and syntenic studies among Cucurbits [34], for downstream applications such as genotyping by sequencing (GBS) [47], and for optical mapping that has been performed in other species to improve draft genome quality [48]. The links established by a more comprehensive anchoring of the melon genome sequence and the genetic map developed here and elsewhere [5,49] will be vital in moving from quantitative trait loci (QTL) for important traits, to cloning of the underlying genes.

The karyotype of the commercially important ‘Piel de Sapo’ Spanish type melon was 2n = 2x = 24 = 16sm + 6ac + 2m (Figure 3A). This was distinct from what has been reported in other types including muskmelon (2n = 2x = 24 = 16m + 4sm + 4stm) [22]; American muskmelon (*Cucumis melo* subsp. *melo* group *reticulatus*) and ‘Hetian’ (*Cucumis melo* subsp. *melo* Pang) as 2n = 2x = 24 = 20m + 4sm and 2n = 2x = 24 = 22m + 2sm, respectively [23]; and two other *C. melo* types, ‘Jiashi’ thick skin and ‘Huangjin’ thin skin type (both with 2n = 2x = 24 = 14m + 10st) [21]. The difference in karyotype may reflect a fundamental difference in the chromosome morphology due to changes in centromere position within the *inodorus* group, or misinterpretation associated with the difficulty of establishing a karyotype with small chromosomes and the lack of a landmark (CentM repeat, for example) to clearly demarcate centromeres. One feature of the PS karyotype was similar to those of other studies, namely the presence of a satellite produced by a secondary constriction in the pericentromeric region on the short arm of CME4 [23]. Large insert DNA clones, in this case BACs, were used for the first time as reliable cytogenetic landmarks to identify the 12 melon chromosomes and in combination with two-color FISH, orient them with respect to the genetic map and PMs (Figure 3B, C, D). Although probes for the 45S and 5S genes and centromere specific repeats were not mapped by FISH to melon chromosomes as in other karyotyping studies [29-31], their positions were inferred after *in silico* analysis. CentSp repetitive sequences, a family of satellite DNAs tandemly arranged in large arrays that are the primary components of the centromeres [29] were located in large zones of reduced recombination and high SNP marker density (Figures 1 and 2). This indicated that, lacking FISH probes for these features, their *in silico* positioning was a good approximation. This work represents the first steps towards the integration of the high-resolution genetic and cytogenetic map with the genomic sequence in melon and provides more information on genome organization.

Although both the number of SNPs obtained and genotyping efficiency can be improved by using an NGS-based genotyping alternative such as GBS [50], we chose the Illumina GoldenGate system in v3.5.1 in order to re-use some of the SNPs and thus have common anchoring points with the previous version v3.5 [9]. Utilizing this system and resequencing data from PS and SC lines, 66% (288/435) of newly designed SNPs called with the simply unified pair-end resequencing (SUPER) pipeline [51] were validated (Additional file 1: Table S2), adding to the more than 1,200 SNP markers validated in the PS x SC population to date (Garcia-Mas, unpublished). The PS line represents the predominant and most economically important melon type grown in Spain and is a parental line in DHL, NIL, and other F2 mapping populations [35,37,52] used to identify QTL for an extensive variety of fruit quality and disease resistance traits [5]. Thus, the additional SNP markers developed here will add to the genotyping resources available for the mapping populations in the same genetic background [53] and facilitate fine mapping and cloning of QTL currently under development [53,54]. Furthermore, despite the fact that newly designed SNPs were selected to be polymorphic between PS and SC, a high percentage can be expected to function in other accessions and outside of the *inodorus* group, as was found for other SNPs developed for the PS x SC cross described in Esteras et al. [19]. The failure rate of SNPs in the GoldenGate assay was higher than in previous studies with melon where validation rates varied between 78 and 91% [9,19]. There was a high correlation between SNP failure and the corresponding size of the unanchored scaffold for which they were designed, illustrating the difficulty of anchoring small scaffolds with highly repetitive sequence, using diminished SNP calling parameters.

Understanding recombination landscape within a species is important for plant breeding applications. A detailed genome-wide and local depiction of recombination rates allows for accurate scaling of map-based cloning projects, marker assisted selection (MAS) strategies for trait introgression, and crossing programs where unfavorable linkage between traits needs to be broken, since regions with high or low recombination rates require correspondingly higher or lower marker densities. At a finer scale, understanding recombination in crop species can help to define recombinationally hyperactive regions or individual genes controlling recombination rate [55] as well as understanding plant genome variability [56]. The genome wide recombination rate calculated in this study (3.3 cM/Mb) for melon was higher than previous (3.1 cM/Mb) [9] primarily reflecting the increased quantity of anchored genomic sequence and correspondingly larger genetic map distances. It was within the range of other cucurbit species, 2.3 cM/Mb and 3.2 cM/Mb for watermelon and cucumber, respectively [17,18]. The recombination rate between chromosomes varied widely, reflecting the size and distribution of recombination suppression zones (Figure 2, Additional file 1: Table S6). For example, positioning of repetitive sequences by BLAST analysis supported the idea that zones of recombination suppression covering large physical distances corresponded to some components of the NOR on LGIV (18S and 5.8S) and LGX (5.8S) as well as the 5S rDNA on LGXII (Additional file 1: Table S7 and Additional file 1: Table S8), as has been reported [30,31,33]. Furthermore, we detected components of NOR on LGI (5.5S, 26S, and 18S), which has not previously been reported. Han et al. [32] detected a large, heterochromatic segment in the pericentromeric region of the long arm of melon chromosome I that accounted for approximately 47% of its length, which may correspond to this zone. Overall, the differences in recombination rates between chromosomes, and the collocation of recombination suppression zones with the genomic scaffolds identified here will aid in the planning for MAS projects and development of mapping populations in melon. For example, it would be necessary to increase the population size to increase the possibilities of obtaining recombinant individuals on LGIV and LGX.

## Conclusions

We provided a significantly improved version of the anchored melon genome by developing a new set of targeted SNP markers with better distribution to construct a high-resolution genetic map. The improved anchoring of the melon genome will permit faster map-based cloning of genes underlying QTL for agronomically important traits, diversity assessment through comparative genomics, and provide important insights on phylogenetic relationships among cucurbits. With the improved anchoring, it was possible to make *in silico* inferences on likely chromosome structure by relating zones of recombination suppression to centromeres and 45S and 5S heterochromatic regions. The FISH mapping with the melon specific BAC probes allowed us to orient the 12 melon chromosomes and develop a karyotype. This represents the first steps towards the integration of the high-resolution genetic and cytogenetic map with the genomic sequence in melon that will provide more information on genome organization and allow for the improvement of the melon genome draft sequence.

## Methods

### Mapping population

An F2 population derived from the cross “Piel de Sapo” T111 (PS) x ‘Songwhan Charmi’ PI 161375 (SC) was grown in winter of 2012. F2 seeds were planted in trays in the greenhouse and leaf samples taken at the 2 leaf stage. DNA was extracted using the CTAB method [57]. DNA concentration was estimated with a Qubit® 2.0 Fluorometer (Life Technologies).

### SNP selection and genotyping

A set of 768 SNPs was selected to perform the new anchoring. These were dived into two sub-sets (Additional file 1: Table S2). The first sub-set was composed of 435 new SNPs selected from the 63 largest unanchored scaffolds of the N98 index predicted to contain up to 40.5 Mb of the scaffold assembly of melon genome version 3.5, or scaffolds that were previously anchored, but unoriented (Additional file 1: Table S1). All SNPs in each unanchored scaffold were first called using the SUPER pipeline [51] based on resequencing data of SC, PS and the reference sequence of DHL92 generated from Illumina paired-end sequencing averaging 70*10^6^ reads per sample at a read length of 150 bp and 22x coverage / 10Gb per sample [9]. SNP calling was done in iterations starting with maximum global quality 999 (phred-scaled quality score for the assertion. -10log_10 prob), minimum read depth of 15x, and absence of other SNPs in 50 bp flanking regions. For smaller scaffolds, it was necessary to decrease the stringency of selection parameters to obtain SNP calls. To increase the likelihood of anchoring and orienting scaffolds, a novel strategy was utilized by selecting at least two SNPs from each scaffold end as determined by their physical position from assembly version 3.5. From 1 to 5 new SNPs were selected per unanchored scaffold, depending on the size. Thus, together with previously validated SNPs, from 1 to 13 SNPs/scaffold were utilized for the re-anchoring. SNPs plus flanking sequences were submitted and evaluated with Illumina Assay Design Tool (ADT) (Illumina, Inc.) and assays selected to have the highest probability of success according to ADT results. The second sub-set contained 333 SNPs previously utilized to anchor the melon genome [9] as detailed in [19]. This second group served as a reference to make inferences across this experiment and the previous genome anchoring study v3.5.

The F2 lines described above were genotyped using the Illumina GoldenGate genotyping assay [58] and a custom 768plex Illumina Golden Gate Panel with Beadarray technology (GoldenGate Universal-32 BeadChips). Results were analyzed in a BeadArray Reader (Illumina, Inc.). SNP genotypes were scored with the Genotyping Module of the GenomeStudio Data Analysis software (Illumina, Inc.) using default parameters. F2 individuals with a call rate ≤ 85% were eliminated from further analysis. Genotype clusters were manually edited when necessary. Data was outputted and entered into the genotyping pipeline as described in [59]. SNPs with GenTrain and GeneCall 10% higher than 0.4 and 0.2, respectively, and at least two genotypic classes were classified as polymorphic and usable. Usable SNPs were then hand inspected and eliminated from the analysis if the genotype of either the PS or SC parent was not unequivocal.

### Genetic map construction and genome anchoring

Following the filtration as described above, the genotyping data was entered into JoinMap v4.1 [60]. Markers were examined by Chi-square analysis and those showing extreme segregation distortions (p = 0.0001) from expected 1:2:1 ratios were eliminated from the analysis. Linkage groups were then calculated using a LOD score of 10 and the ML mapping algorithm with default parameters. Further pruning of the data within LGs was conducted by eliminating individuals with >10% missing genotypes. Markers with segregation ratios that differed at p = 0.05 were classified as displaying segregation distortion. To anchor the genome assembly to the genetic map, BLASTN analysis was performed with e-value cutoff of 1E-30 for every SNP marker in the genetic map against the genome assembly. Scaffolds were then assigned to linkage groups accordingly. When more than one marker had hits on the same scaffold, it was possible to orientate this scaffold on the map. A small group of markers that were not located consecutively on the genetic map according to their physical location in a scaffold were eliminated from further analysis when there were more than two other markers with recombination to accurately establish the orientation of a scaffold (Additional file 1: Table S1). For scaffolds that were not oriented, information from the genetic map of the first version of the melon genome anchoring was used as a reference [9]. The genome anchoring to the pseudomolecules was drawn with the Harry Plotter software [61].

### Correction of genome assembly and construction of chromosome-scale pseudomolecules

Manual correction of 6 chimeric scaffolds consisted of splitting them in two separate scaffolds, with the splitting point selected between the two contigs that showed most inconsistencies in paired-end links. The chosen cut point was located in Ns tracks, except in CM3.5_scaffold00063. An error in the cut point of CM3.5_scaffold01599, which was originally created by splitting CM3.5_scaffold00056 [9], was also identified. The 5′ split fragment of the former was re-joined to the 3′ end of the latter. According to the order and orientation of the scaffolds in the new physical map and the corrections made in the genome assembly, scaffolds were ordered into chromosome-scale PMs where each scaffold was joined by a 1,000 N track. The non-anchored scaffolds and contigs were joined in the non-ordered chromosome zero, also joined by a 1,000 N track. A new gff3 file with new gene coordinates in corrected scaffolds was also obtained. All these files can be accessed at http://www.melonomics.net/files/Genome/Melon_genome_v3.5.1.

### Estimation of recombination

The average genome-wide recombination rate (GWRR) and the average recombination rate for each chromosome were expressed as the ratio of total genetic map length in centiMorgans divided by the genome size and physical length of the chromosome in megabase pairs, respectively. Recombination rates along each LG were estimated by first plotting Marey maps of the genetic positions of molecular markers (in centimorgans, cM) against their physical position (in Megabase pairs, Mb). Cumulative recombination curves for each chromosome were then estimated using a cubic spline method (implemented in the standard library of R, http://cran.r-project.org) and a sliding window of 1 Mb. The recombination value per position was obtained calculating the slope per window (that is, the derivative) and their curves were plotted for each of the chromosomes.

### Demarcating centromeres and pericentromeric boundaries of melon pseudomolecules

The putative locations of the centromere of melon PMs were identified by aligning *C. melo* DNA for the 352 bp centromere specific pSat107 satellite (pSat107) [GI:3929695] [62] and these clustered sequences: *Cucumis melo* centromere-specific repeat A sequence (CentSpA) [GI:212961153], *Cucumis melo* centromere-specific repeat B sequence (CentSpB) [GI:212961171] and *Cucumis melo* centromere-specific repeat C sequence (CentSpC) [GI:212961196] [29] to the melon genome assembly v3.5.1 with blastn (version: blast 2.2.27). We retained the alignments that co-localized 2 or more different centromeric-specific sequences (pSat107 and any of CentSp), with a coverage of 80% or more and sequence identity of 80% or more. Putative locations of 45S and 5S rDNA were identified using the ribosomal gene annotations obtained previously [9] and by supplementing this data with RNAmmer predictions [http://www.cbs.dtu.dk/services/RNAmmer/] [63] with the default parameters.

### Cytogenetics - plant material and chromosome preparation for cytogenetic analysis

PS seeds were germinated in Petri dishes. Root tips were cut, directly fixed in methanol/acid acetic (3:1) and subsequently digested in 4% (wt/vol) cellulose R-10 Onozuka, 1% (wt/vol) pectolyase Y23 and 4% (wt/vol) hemicellulase in 0.01 M citrate buffer (1/1 volume of 0.01 M citric acid and 0.01 M tri-sodium citrate) for one hour at 37°C. Enzyme solution was removed and tips were washed two times in 0.01M citrate buffer for 10 minutes at room temperature. The material was centrifuged during 5 minutes at 4,000 rpm and the resulting pellet was diluted in fresh ethanol/acid acetic (3:1). Slides were air dried at room temperature and stored at −20°C until use.

### Probe preparation and Fluorescent in situ Hybridization (FISH)

BAC clones for FISH analysis (Table 2) were selected from an existing 23,040 BAC library from the double-haploid melon line DHL92 described previously [64]. BACs were previously anchored by SSR, SNP, and RFLP markers that mapped on extreme ends of each of the 12 melon LGs [49]. The positions of anchored markers on the genetic map were then used to establish a link between the scaffold-based physical map and the cytogenetic map. BLASTN analysis was conducted against the melon genome assembly (http://www.melonomics.net/tools/blast/run/) on BAC end sequences when available or marker sequences, to ensure that they were located in the expected LG and present in only a single copy in the genome. DNA from BACs was extracted according to standard protocols using the QIAGEN Plasmid kit (QIAGEN), and a PCR with specific primers was performed to confirm the correct selection of each BAC clone before FISH analysis.

Fluorescence in situ hybridization (FISH) with specific BAC clones was performed on metaphase chromosomes as previously described [65] with modifications. Briefly, 1 μg of the BAC DNA was labelled with digoxigenin-11-dUTP or biotin-11-dUTP by Nick Translation technique (Abbot kit) and ethanol precipitated with competitor DNA (melon genomic DNA), salmon sperm DNA (Invitrogen, 10 mg/ml) and 1/10 volume of 3 M sodium acetate overnight at −20°C. For two-colour FISH, two different probes were labelled with digoxinenenin-11-dUTP and biotin-11-dUTP, respectively. About 250 ng of labelled DNA was used for each hybridization experiment. The precipitated probe mix was resuspended in 14 μl hybridization buffer (50% deionised formamide, 10% dextran sulfate, 2xSSC and 0.5 M sodium phosphate), denaturated 80°C for 10 min and preannealed at 37°C for 1 h. After overnight hybridization, slides were washed two times in 50% formamide/2xSSC at 42°C for 10 min followed by three washes in 2xSSC at 42°C for 5 min each. Chromosomes were counterstained with DAPI (4′, 6-diamidino-2-phenylindole) in Vectashield antifade.

### Microscopy

Preparations were visualized using a Zeiss Axioskop epifluorescence microscope equipped with the appropriate filters and a charged coupled device camera (ProgRes® CS10plus, Jenoptik). Chromosomes were ordered in a standard karyotypic layout, with the short arms on top. For simplicity, we decided not to change the alignment and orientation of LGs and PMs to match the orientation of chromosomes of the karyotype, but to conserve it according to the standard nomenclature and orientation of melon LGs established in Périn et al. [66]. The three-letter acronym CME was used to designate the genus and species (*Cucumis melo*), followed by the Arabic numeral that denotes the chromosome number.

### Availability of supporting data

Anchored scaffold and pseudomolecule sequences are deposited in the European Nucleotide Archive (ENA) and can be accessed at http://www.ebi.ac.uk/ena/data/view/PRJEB68 with accession numbers LN681792-LN713266. The v3.5.1 sequences can also be found at http://melonomics.net/*.* All other supporting data are included as additional files.

## Additional files

Additional file 1: Table S1. Largest scaffolds of melon genome assembly v3.5. **Table S2.** 768 SNPs selected to reanchor melon genome assembly 3.5. **Table S3.** Segregation distortion in the PS x SC F2 mapping population. **Table S4.** Chimeric and misassembled scaffolds identified in assembly v3.5. **Table S5.** Scaffolds of assembly v3.5.1 anchored to the genetic map. **Table S6.** Recombination frequencies along 12 melon PMs measured in 1 Mb windows. **Table S7.** BLAST analysis with four distinct centromere specific repeats: sSat107 and CentSpA, B, C. **Table S8.** BLAST predictions of locations of 45S and 5S rDNA in melon scaffolds using ribosomal gene annotations obtained previously (Garcia-Mas et al. [9]) and by supplementing with RNAmmer predictions [http://www.cbs.dtu.dk/services/RNAmmer/]. **Table S9.** Metrics of recently sequenced plant genomes. Numbers in parentheses are following reanchoring of genome. Additional file 2: Figure S1. Fluorescence in situ hybridization (FISH) with BAC clones to melon metaphase chromosomes labelled with digoxinenenin-11-dUTP (green) or biotin-11-dUTP (red). Additional file 3: Figure S2. Percentage of the scaffold genome assembly anchored as a function of the number of scaffolds. Vertical line represents scaffolds contained in the N98 index anchored in this study.

## Abbreviations

NGS: Next generation sequencing
WGS: Whole-genome shotgun
SNP: Single nucleotide polymorphism
PM: Pseudomolecule
DHLs: Doubled haploid lines
FISH: Fluorescence in situ hybridization
BACs: Bacterial artificial chromosome
YAC: Yeast artificial chromosome
PS: Piel de Sapo
SC: Songwhan charmi
LG: Linkage group
EST: Expressed sequence tag
MAS: Marker assisted selection
NOR: Nucleolus organizer region
GBS: Genotyping-by-sequencing
