# Supplementary figures and images for: Use of targeted SNP selection for an improved anchoring of the melon (Cucumis melo L.) scaffold genome assembly
Source: BMC Genomics. 2015 Jan 22;16(1):4. doi: 10.1186/s12864-014-1196-3 (PMC4316794; doi:10.1186/s12864-014-1196-3)

LG I

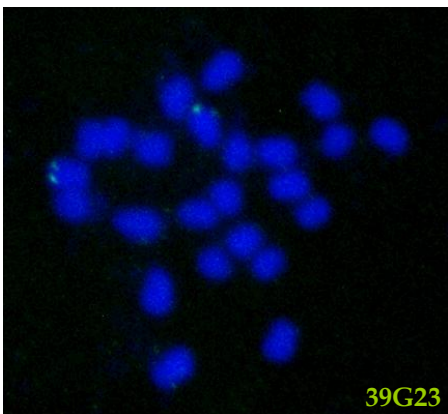

LG II

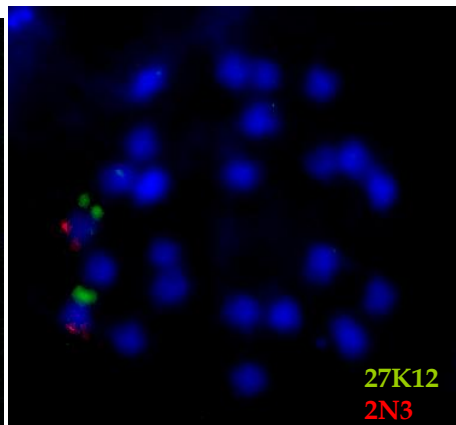

LG III

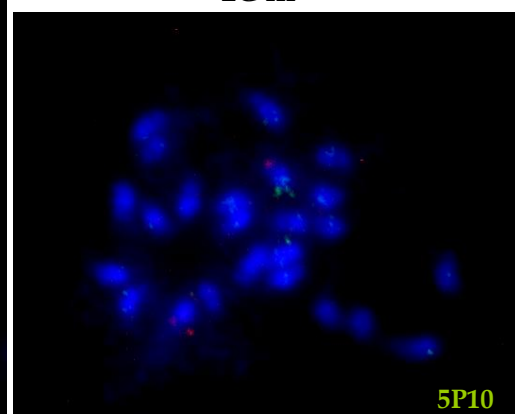

LG IV

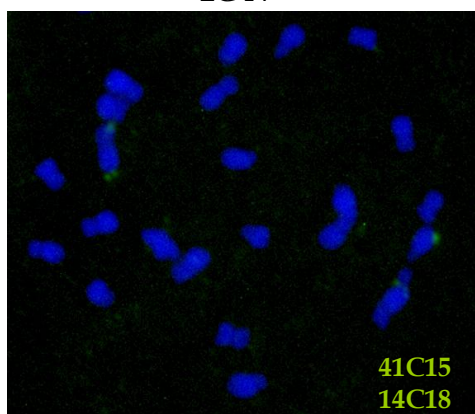

LG V

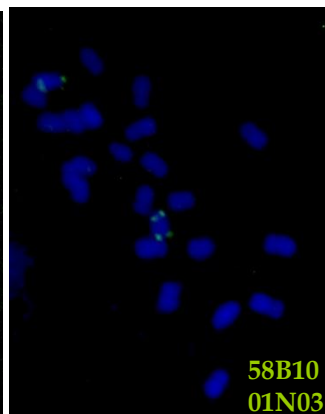

LG VI

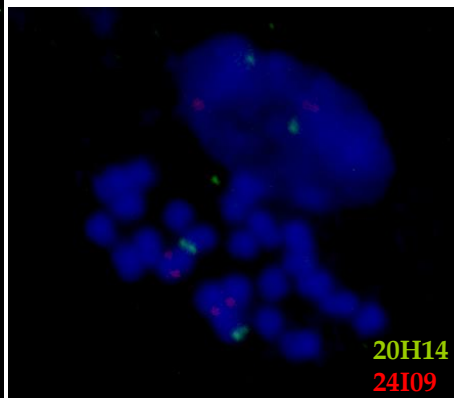

LG VIII

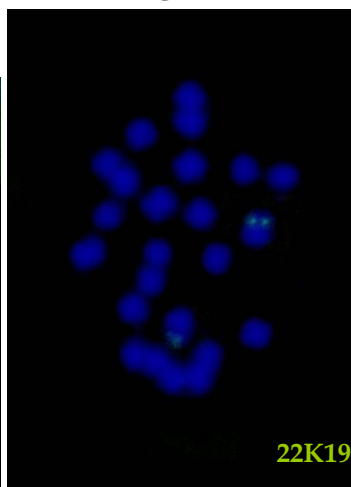

LG VII

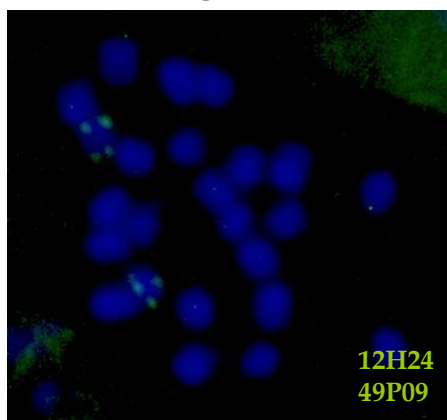

LG IX

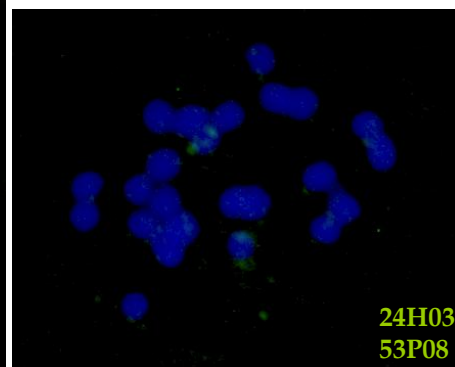

LG X

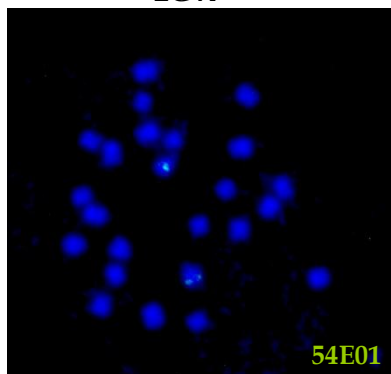

LG XI

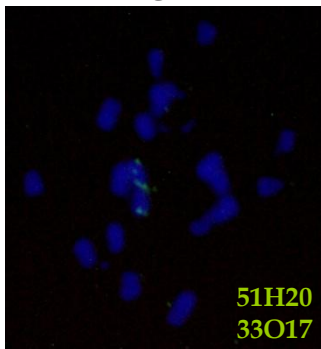

LG XII

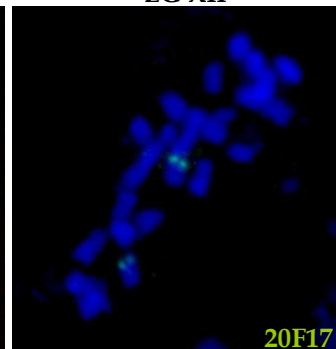

Supplement: Additional file 2: Figure S1. — Fluorescence in situ hybridization (FISH) with BAC clones to melon metaphase chromosomes labelled with digoxinenenin-11-dUTP (green) or biotin-11-dUTP (red). [file 12864_2014_1196_MOESM2_ESM.pdf]

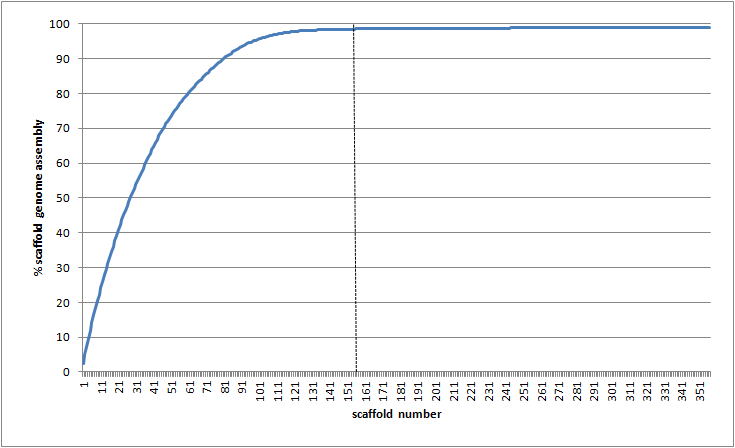

Supplement: Additional file 3: Figure S2. — Percentage of the scaffold genome assembly anchored as a function of the number of scaffolds. Vertical line represents scaffolds contained in the N98 index anchored in this study. [file 12864_2014_1196_MOESM3_ESM.docx]
